# Greener Selective Cycloalkane Oxidations with Hydrogen Peroxide Catalyzed by Copper-5-(4-pyridyl)tetrazolate Metal-Organic Frameworks

**DOI:** 10.3390/molecules201019203

**Published:** 2015-10-21

**Authors:** Luísa Martins, Rajendar Nasani, Manideepa Saha, Shaikh Mobin, Suman Mukhopadhyay, Armando Pombeiro

**Affiliations:** 1 Chemical Engineering Departament, Instituto Superior de Engenharia de Lisboa, Instituto Politécnico de Lisboa, R. Conselheiro Emídio Navarro, 1959-007 Lisboa, Portugal; 2 Centro de Química Estrutural, Complexo I, Instituto Superior Técnico, Universidade de Lisboa, Av. Rovisco Pais, 1049-001 Lisboa, Portugal; 3 Department of Chemistry, School of Basic Sciences, Indian Institute of Technology Indore, IET-DAVV Campus, Khandwa Road, Indore 452017, India; E-Mails: nrajendra@iiti.ac.in (R.N.); phd11113104@iiti.ac.in (M.S.); xray@iiti.ac.in (S.M.)

**Keywords:** efficient cycloalkane oxidation, reusable heterogeneous catalysts, Cu(I or II) MOFs, microwave, solvent-free, green oxidation

## Abstract

Microwave assisted synthesis of the Cu(I) compound [Cu(µ_4_-4-ptz)]*_n_* [**1**, 4-ptz = 5-(4-pyridyl)tetrazolate] has been performed by employing a relatively easy method and within a shorter period of time compared to its sister compounds. The syntheses of the Cu(II) compounds [Cu_3_(µ_3_-4-ptz)_4_(µ_2_-N_3_)_2_(DMF)_2_]*_n_*∙(DMF)_2*n*_ (**2**) and [Cu(µ_2_-4-ptz)_2_(H_2_O)_2_]*_n_* (**3**) using a similar method were reported previously by us. MOFs **1**-**3** revealed high catalytic activity toward oxidation of cyclic alkanes (cyclopentane, -hexane and -octane) with aqueous hydrogen peroxide, under very mild conditions (at room temperature), without any added solvent or additive. The most efficient system (**2/**H_2_O_2_) showed, for the oxidation of cyclohexane, a turnover number (TON) of 396 (TOF of 40 h^−1^), with an overall product yield (cyclohexanol and cyclohexanone) of 40% relative to the substrate. Moreover, the heterogeneous catalytic systems **1**–**3** allowed an easy catalyst recovery and reuse, at least for four consecutive cycles, maintaining *ca.* 90% of the initial high activity and concomitant high selectivity.

## 1. Introduction

**Scheme 1:**
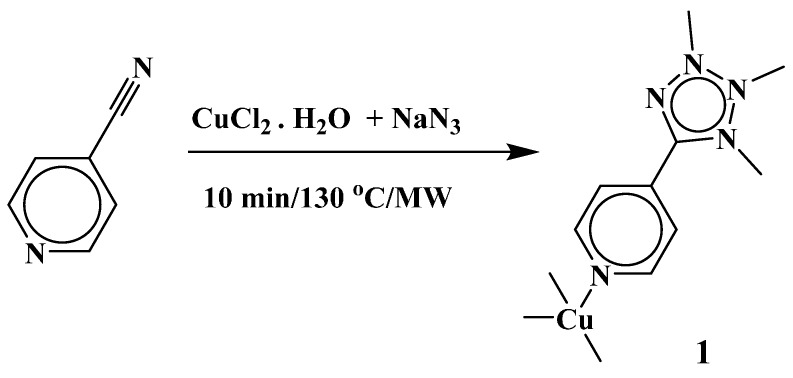
Synthesis of MOF compound [Cu(µ_4_-4-ptz)]*_n_* (**1**).

**Scheme 2:**
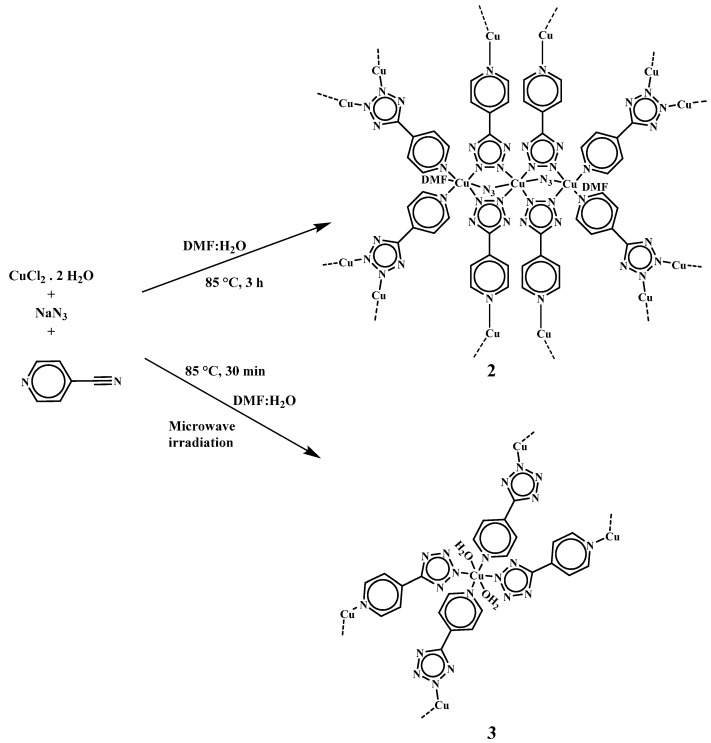
Synthetic procedures of compounds [Cu_3_(µ_3_-4-ptz)_4_(µ_2_-N_3_)_2_(DMF)_2_]*_n_*∙(DMF)_2*n*_ (**2**) and {[Cu(µ_2_-4-ptz)_2_(H_2_O)_2_]}*_n_* (**3**) [8].

In the last decade, 5-substituted tetrazole ligands have been evolving as one of the more useful linkers for generation of functional materials due to their interesting chemical and structural properties. A few methods have been followed until now for *in-situ* tetrazole generation by cycloaddition between an organonitrile and an azide in the presence of a transition metal ion, where the solvothermal process has dominated to a large extent over the other ones [1,2,3]. Although there are a few reports where tetrazoles had been prepared under mild conditions in the presence of metal ions and other catalysts [4,5,6,7], descriptions of construction of a MOF containing 5-(4-pyridyl)tetrazolate (4-ptz) building blocks in a controlled manner other than those involving a solvothermal process (at lower temperatures and pressures via a similar cycloaddition path) are scarce. In the present study, a highly improved synthesis of **1** is reported (Scheme 1), whereas the syntheses of **2** and **3** (Scheme 2) had previously been reported by us [8]. These compounds were generated while studying the effect of reaction conditions on the coordination modes of 4-pytz by employing the [2 + 3] cycloaddition as a tool for generating the 5-substituted tetrazole ligands *in-situ* from 4-pyridinecarbonitrile and NaN_3_ in the presence of a copper(II) salt. Curiously, though there is a report with a structure similar to **1** (which was produced via the solvothermal method in the time period of one day) [3], in our case the time has been significantly reduced to 10 min by using microwave irradiation. This procedure can be considered as an easy to handle and relatively energy efficient method for the production of Cu(I) MOFs in pure form. It is interesting to note that, to our knowledge, this is the first procedure where a MOF is generated by a one-pot reduction of Cu(II) to Cu(I) and formation of a tetrazolate linker via [2 + 3] cycloaddition within such a short time (10 min).

Although copper based metal-organic materials as catalysts for hydrocarbon oxidation have been studied in the last few years with diverse ligand systems [9,10,11,12,13,14,15,16,17,18], the application of tetrazole based copper-organic frameworks for the oxidation of alkanes under mild oxidation conditions remains largely unexplored.

Selective partial oxidation of alkanes is an important topic with potential in terms of economic and ecological perspectives of sustainable chemistry. However, efficient catalytic oxidation of alkanes still remains as a challenging topic. A relevant example of selective alkane oxidation [9,19,20,21,22,23,24,25,26,27] with industrial significance concerns the oxidation of cyclohexane to cyclohexanol and cyclohexanone that are important reagents for the production of adipic acid and caprolactam used for the manufacture of nylon [19,20,21,27]. The current industrial route uses a homogeneous cobalt species as catalyst, dioxygen as oxidant and requires considerably harsh conditions (150 °C), forming the oxidation products in low yields (*ca*. 5%) to achieve a good selectivity (*ca*. 85%), [19,20,21]. The development of more efficient catalysts, under milder conditions, at room temperature and using low toxicity media and oxidizing agents is needed [18,19,20,21,27,28,29,30,31,32,33,34]. Hydrogen peroxide is one of the best options in this regard since H_2_O is the sole by-product and exhibits an atom efficiency and e-factor similar to dioxygen [34]. Thus, peroxidative (with H_2_O_2_) alkane oxidations were the selected reactions for the present study. Herein we report the catalytic performances of Cu(I) 1 and Cu(II) **2** and **3** compounds toward the oxidation of cyclic alkanes (cyclopentane, -hexane and -octane) under very mild and green (solvent- and additive-free) conditions as a significant step towards the protection of environment and quality of life.

## 2. Results and Discussion

### 2.1. Synthesis and Spectroscopic Characterization of [Cu(µ_4_-4-ptz)]_n_ (**1**)

Compound **1** was prepared by microwave irradiation, at 130 °C for 10 min, of a mixture containing copper(II) chloride, sodium azide and 4-cyanopyridine in 1:2:4 molar ratios using a water-DMF mixture (1:6, *v*:*v*), from which the yellow colored Cu(I) compound **1** crystallized upon cooling to room temperature.

**Figure 1:**
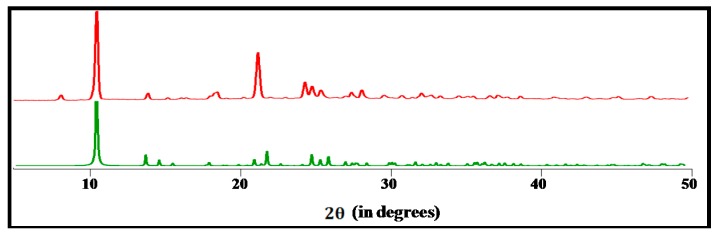
PXRD patterns of **1**, simulated (green) and bulk sample (red).

The obtained compound was characterized by IR spectroscopy, elemental analysis and single-crystal X-ray crystallography. The IR spectrum of **1** shows a strong band at 1650 cm^−1^ [35,36] indicating the presence of the tetrazolate moiety. The SCXRD data of compound **1** (Figure 1) revealed that its structural properties are similar to those of the reported compound [3]. Powder X-ray diffraction (PXRD) patterns recorded for bulk samples of **1** (Figure 1) show a very good matching with the respective simulated patterns (acquired from the single crystal X-ray data) thus demonstrating their phase purity.

**Figure 2:**
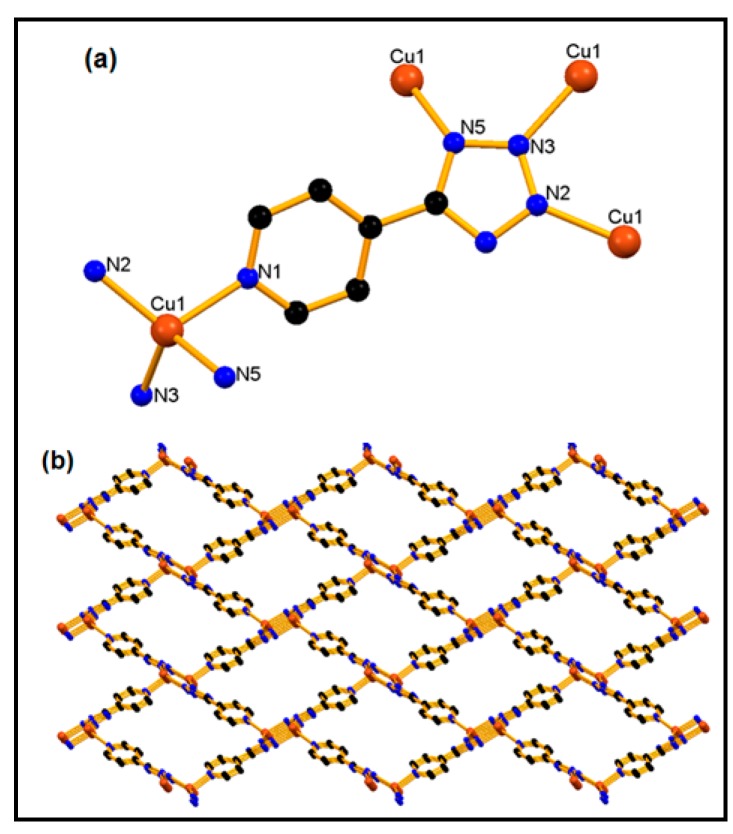
Structural fragments of **1** representing (**a**) the basic unit; (**b**) a view of rhomboid voids along the *a*-axis. Color codes: Cu brown, C black, and N blue.

Single crystal X-ray diffraction studies indicate that **1** had grown as a rigid three-dimensional framework with one-dimensional open-ended pores. In this framework Cu(I) is acting as the central metal ion. It is coordinated by four nitrogen atoms of four 4-ptz ligands, among them one atom being the pyridine nitrogen (N1) and the remaining three atoms pertaining to the tetrazolate ring (N2, N3 and N5) from four 4-ptz linkers, and exhibits a distorted tetrahedron geometry (Figure 2a). Each 4-ptz is coordinated to four different metal centers and is acting as a tetradentate ligand.

### 2.2. Catalytic Oxidation of Cycloalkanes

**Scheme 3:**
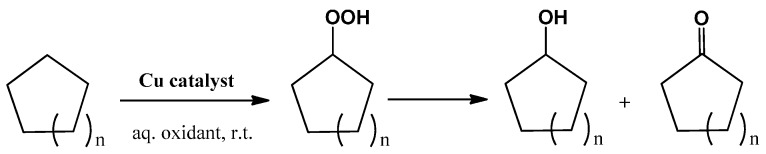
Solvent-free oxidation of cycloalkanes (*n* = 1, 2 or 4) to the corresponding alcohol and ketone mixtures.

Complexes **1**–**3** were tested as catalysts for the oxidation of cyclic alkanes (cyclopentane, -hexane and -octane) to the corresponding alcohol and ketone (the final products) mixtures via formation of cycloalkyl hydroperoxide (CyOOH, primary product) [31,37,38,39,40], according to Scheme 3 and Table 1 and Table 2 (see below). The catalytic systems are based on any of the above Cu(I) **1** or Cu(II) **2** or **3** complexes, hydrogen peroxide (30% aqueous solution) as the oxidizing agent, at room temperature (r.t.), in the absence of added solvent or additives. The use of other environmentally benign [34] peroxidative oxidants, such as *tert*-butyl hydroperoxide (TBHP, 70% aqueous solution), was also considered.

**Table 1:** Oxidation of selected cycloalkanes using **1**–**3** as catalysts (selected data) ^a^.

Entry	Catalyst	Substrate	Oxidant	TON [TOF (h^−1^)] ^b^	OL	Yield (%) ^c^ ONE	Total ^d^	OL/ONE Ratio
1	**1**	cyclopentane	H_2_O_2_	260 (26)	14.3	11.7	26.0	1.2
2			TBHP	440 (44)	5.5	16.7	22.2	0.3
3		cyclohexane	H_2_O_2_	369 (37)	18.8	18.1	36.9	1.0
4			TBHP	664 (66)	11.3	21.9	33.2	0.5
5		cyclooctane	H_2_O_2_	218 (22)	11.7	10.1	21.8	1.2
6			TBHP	434 (43)	8.3	13.4	21.7	0.6
7	**2**	cyclopentane	H_2_O_2_	276 (28)	13.1	14.5	27.6	0.9
8			TBHP	416 (42)	7.6	13.2	20.8	0.6
9		cyclohexane	H_2_O_2_	396 (40)	20.8	18.8	39.6	1.1
10			TBHP	704 (70)	12.9	22.3	35.2	0.6
11		cyclooctane	H_2_O_2_	343 (34)	21.4	12.9	34.3	1.7
12			TBHP	620 (62)	5.3	25.7	31.0	0.2
13	**3**	cyclopentane	H_2_O_2_	215 (22)	11.9	9.6	21.5	1.2
14			TBHP	218 (22)	4.0	6.9	10.9	0.6
15		cyclohexane	H_2_O_2_	366 (37)	16.5	20.1	36.6	0.8
16			TBHP	710 (71)	13.9	21.6	35.5	0.6
17		cyclooctane	H_2_O_2_	285 (29)	17.8	10.7	28.5	1.7
18			TBHP	468 (47)	8.7	14.7	23.4	0.6
19	none	cyclopentane	H_2_O_2_	-	2.1	1.1	3.2	1.9
20			TBHP	-	1.1	1.8	2.9	0.6
21		cyclohexane	H_2_O_2_	-	2.4	1.4	3.8	1.7
22			TBHP	-	1.3	2.5	3.8	0.5
23		cyclooctane	H_2_O_2_	-	2.2	1.5	3.7	1.5
24			TBHP	-	0.7	1.3	2.0	0.5

^a^ Reaction conditions unless stated otherwise: 5.0 mmol of substrate, 2.5–5 μmol of catalyst, 10.0 mmol of oxidant, r.t., 10 h reaction time. Yield and TON determined by GC analysis (upon treatment with PPh_3_). ^b^ Turnover number = number of moles of products per mol of catalyst; TOF = TON per hour (values in brackets). ^c^ Molar yield (%) based on substrate, *i.e.*, moles of product (alcohol (OL) or ketone (ONE)) per 100 mol of cycloalkane. ^d^ Moles of alcohol + ketone per 100 moles of cyclohexane.

The formation of CyOOH (under the conditions of Table 1) is proved by using the method proposed by Shul’pin [37,38,39,40]. The addition of PPh_3_ prior to the GC analysis of the products results in a marked increase of the amount of alcohol (due to reduction of CyOOH by PPh_3_, with formation of phosphane oxide) and a corresponding decrease of ketone, as observed in other catalytic systems [9,10,11,12,13,41,42,43,44,45].

**Table 2:** Peroxidative oxidation of cyclohexane with H_2_O_2_ (selected data) ^a^.

Entry	Catalyst	*n*(cat.)/*n*(CyH) × 10^3^	*n*(H_2_O_2_)/*n*(cat.) × 10^−3^	Reaction Time (h)	Yield (%) ^b^			TON [TOF (h^−1^)] ^c^
					OL	ONE	Total ^d^	
1	**1**	4	0.5	10	11.1	6.7	17.8	45 (4.5)
2		2	1	10	16.6	11.5	28.1	141 (14)
3		1.3	1.5	10	16.2	17.8	34.0	262 (26)
4		0.8	2.5	10	12.4	21.9	34.3	429 (43)
5		0.4	5	10	2.1	18.0	19.9	498 (50)
6		1	2	0.25	5.5	2.6	8.1	81 (8.1)
7		1	2	0.5	8.4	2.6	11.0	110 (11)
8		1	2	1	9.1	5.8	14.9	149 (15)
9		1	2	2.5	10.5	11.3	21.8	218 (22)
10		1	2	5	17.1	13.7	30.8	308 (31)
11		1	2	12.5	18.7	16.0	34.7	347 (35)
12		1	2	24	6.0	19.3	25.3	253 (25)
13 ^e^		1	2	10	1.3	1.9	3.2	32 (3.2)
14 ^f^		1	2	10	2.8	2.3	5.1	51 (5.1)
15 ^g^		1	2	10	1.1	0.2	1.3	13 (1.3)
16 ^h^		1	2	10	1.6	1.1	2.7	27 (2.7)
17 ^i^		1	2	10	9.3	25.0	34.3	343 (34)
18	**2**	4	0.5	10	10.2	5.4	15.6	39 (4)
19		2	1	10	17.6	10.8	28.4	142 (14)
20		1.3	1.5	10	16.7	20.3	37.0	285 (29)
21		0.8	2.5	10	16.9	19.5	36.4	455 (46)
22		0.4	5	10	5.8	10.7	16.5	413 (41)
23		1	2	0.25	7.5	2.4	9.9	99 (9.9)
24		1	2	0.5	10.6	3.3	13.9	139 (14)
25		1	2	1	14.4	4.9	19.3	193 (19)
26		1	2	2.5	19.7	8.8	28.5	285 (29)
27		1	2	5	17.8	18.1	35.9	359 (36)
28		1	2	12.5	18.0	19.6	37.6	376 (38)
29		1	2	24	5.5	21.7	27.2	272 (27)
30 ^e^		1	2	10	0.6	0.7	1.3	13 (1.3)
31 ^f^		1	2	10	1.1	3.5	4.5	45 (4.5)
32 ^g^		1	2	10	1.7	0.8	2.5	25 (2.5)
33 ^h^		1	2	10	0.9	1.2	2.1	21 (2.1)
34 ^i^		1	2	10	11.9	26.0	37.9	379 (38)
35	**3**	4	0.5	10	10.2	8.7	18.9	47 (4.7)
36		2	1	10	17.1	12.9	30.0	150 (15)
37		1.3	1.5	10	16.3	18.8	35.1	270 (27)
38		0.8	2.5	10	15.2	20.1	35.3	441 (44)
39		0.4	5	10	8.4	15.3	23.7	593 (59)
40		1	2	0.25	7.1	4.4	11.5	115 (12)
41		1	2	0.5	8.3	5.7	14.0	140 (14)
42		1	2	1	9.1	8.5	17.6	176 (18)
43		1	2	2.5	15.6	11.9	27.5	275 (28)
44		1	2	5	19.5	14.1	33.6	336 (34)
45		1	2	12.5	22.9	20.2	35.1	351 (35)
46		2	1	24	14.9	40.1	23.5	235 (24)
47 ^e^		1	2	10	0.8	0.6	1.4	14 (1.4)
48 ^f^		1	2	10	1.2	1.1	2.3	23 (2.3)
49 ^g^		1	2	10	1.4	0.7	2.1	21 (2.1)
50 ^h^		1	2	10	0.7	1.2	1.9	19 (1.9)
51 ^i^		1	2	10	8.4	26.9	35.3	353 (35)

^a^ Reaction conditions (unless stated otherwise): cyclohexane (5.0 mmol), 2–20 μmol of **1**–**3**, H_2_O_2_ (10 mmol), r.t., 0.25–24 h reaction time. Percentage of yield, TON determined by GC analysis (upon treatment with PPh_3_). ^b^ Molar yield (%) based on substrate, *i.e.*, moles of products (cyclohexanol (OL) or cyclohexanone (ONE)) per 100 mol of cyclohexane. ^c^ Turnover number = moles of products per mol of catalyst; TOF = TON per hour (values in brackets). ^d^ Moles of cyclohexanol + cyclohexanone per 100 moles of cyclohexane. ^e^ Reaction in the presence of nitric acid. ^f^ Reaction in the presence of Hpca. ^g^ Reaction in the presence of CBrCl_3_ (5.0 mmol). ^h^ Reaction in the presence of Ph_2_NH (5.0 mmol). ^i^ values from GC analysis prior to addition of PPh_3_ (for comparative purposes).

**Figure 3:**
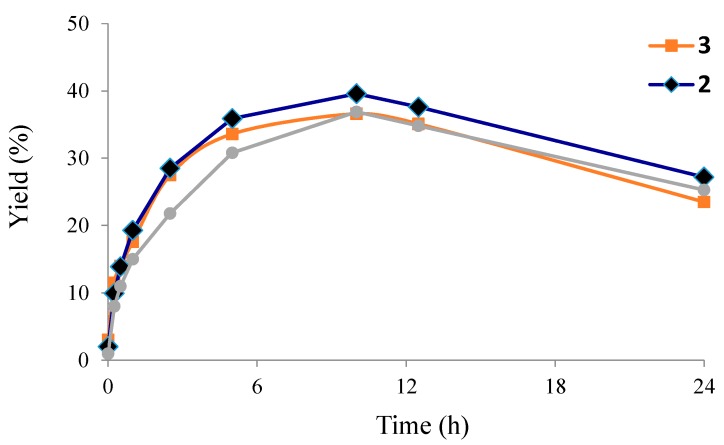
Dependence of the overall yield (mol %, based on substrate) of the products (cyclohexanol + cyclohexanone) on the reaction time, for the oxidation of cyclohexane. Reaction conditions: cyclohexane (5.0 mmol), 5.0 μmol of **1** (•), **2** (♦) or **3** (■), *n*(H_2_O_2_)/*n*(catalyst) (2 × 10^3^), r.t.

Complex **2** provides the most efficient catalytic system, achieving overall yields (relative to the alkane) up to 39.6%, 34.3% and 27.6% for the peroxidative (with H_2_O_2_) oxidation of cyclohexane, -octane and -pentane, respectively (Table 1, entries 9, 11 and 7, respectively) after 10 h reaction time (Figure 3) which can conceivably be due to the presence of azide ligands. Their basic character could promote proton-transfer steps, a feature that is favorable to the occurrence of oxidation catalysis with peroxides. The highest yields were obtained for cyclohexane oxidation (39.6%, 36.9% and 33.6% for **2**, **1** and **3**, entries 9, 3 and 15, Table 1, respectively).

In general, the need of lower catalyst loads (0.05 mol% *vs.* substrate) and, thus, higher turnover numbers (TONs, number of moles of products per mol of catalyst) were found using TBHP (up to 710, Table 1). Moreover, TBHP appears to favor the formation of ketone (ratios alcohol/ketone equal or lower than 0.6, Table 1). The higher activity of this oxidant is also apparent on the over-oxidation products detected by GC-MS (mainly 1,2-cyclohexanediol and 1,4-cyclohexanedione) when the reaction was run in its presence.

Blank tests were performed under the same reaction conditions (Table 1) but in the absence of MOFs and no significant conversion of the cycloalkanes was observed (entries 19–24, Table 1). Moreover, the replacement of **1**–**3** by their precursor salt CuCl_2_·2H_2_O, resulted in a drastic decrease of activity (maximum total yields of 4, 5 and 5%, respectively for cyclopentane, -hexane and -octane), suggesting a significant role of the ligands in the catalytic oxidation of the tested alkanes.

It should be emphasized that yields approaching 40% obtained herein for cyclohexane oxidation can be considered as remarkably high for the oxidation of very inert alkanes, much above those reported for other copper(II) complexes (e.g., bearing *N*,*O*-ligands such as aminopolyalcohols, scorpionates or derivatives [45,46,47], functionalized azo derivatives of β-diketones [17] or Schiff bases), although the values of the present work are obtained at higher reaction times (10 h instead of the usual 6 h). This can conceivably be due to a lower activity toward further oxidation of the alcohol/ketone mixture of **1**–**3** that avoid the over oxidation usually reported for the other Cu systems, or may result from a longer lifetime of our catalysts.

The obtained yield is also much higher than that of the industrial process [19,21,27] in spite of the used mild conditions [ambient temperature, atmospheric pressure, with an aqueous green oxidant, with considerable low loads of catalyst (up to 0.2 mol % of Cu catalyst *vs.* substrate) and without the addition of any solvent or additive].

Moreover, a high selectivity towards the formation of the alcohol/ketone mixtures is exhibited by our systems, since no traces of by-products were detected by GC-MS analysis of the final reaction mixtures for the optimized conditions. These features are of utmost importance for the establishment of a greener catalytic process for cyclohexane oxidation.

**Figure 4:**
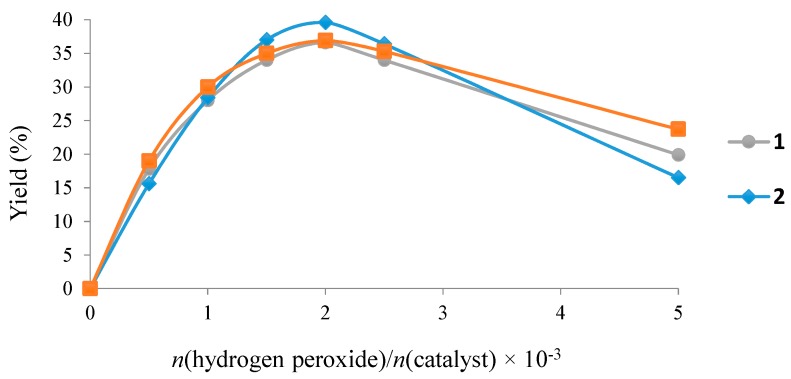
Dependence of the overall yield (mol %, based on substrate) of the products (cyclohexanol + cyclohexanone) on the amount of oxidant (H_2_O_2_, molar ratio relatively to **1** (•), **2** (♦) or **3** (■)) in the oxidation of cyclohexane. Reaction conditions: *n*(H_2_O_2_)/*n*(catalyst) (0–5 × 10^3^), cyclohexane (5.0 mmol), r.t., 10 h.

The influence of various reaction parameters, such as time, the amounts of catalyst and oxidant and presence of additives were investigated for the most active substrate (cyclohexane)/oxidant (H_2_O_2_) system and the results are summarized in Table 2 and Figure 3 and Figure 4.

The yield drop observed (Figure 3) for reaction times higher than 10 h results from the occurrence of subsequent reactions in the oxidative medium. Over-oxidation products such as 1,3-cyclohexanediol and 1,4-cyclohexanediol were detected by CG-MS for 24 h reaction time.

The effect of the peroxide-to-catalyst molar ratio is depicted in Figure 4. The increase of the peroxide amount up to then (H_2_O_2_)/*n*(catalyst) molar ratio of 2 × 10^3^ leads to the maximum products yields. Further increase of the oxidant amount results in a marked yield drop due to over oxidation reactions at higher H_2_O_2_ amounts. In fact, the adipic acid precursor 1,2-cyclohexanediol was detected by GC-MS using the conditions of entry 22, Table 2.

The previously recognized usual promoting effect of an inorganic acid [11,12,13,45,46,47,48,49,50,51,52,53,54] or pyrazinecarboxylic acid (Hpca) [43,51,52,53,54,55,56,57] on the peroxidative oxidation of alkanes catalyzed by homogeneous [27,31,32,43,48,51,52,54,56] or supported [51,52,53,54,56] metallic species is not observed for the present systems. Moreover, the presence of acid (either mineral or organic) has a strong inhibitor effect of the catalytic activity (Table 2, entries 13, 14, 30, 31, 47 or 47). A similar behavior was found for C-scorpionate Au(III) complexes [56]. 

As observed for other Cu and different metal catalytic systems [9,10,11,13,17,37,45,46,47,49,50,51,54,56,57], introduction of a radical trap (CBrCl_3_ or Ph_2_NH) into the reaction mixture results in a considerable suppression of the catalytic activity. This behavior, along with the formation of cyclohexyl hydroperoxide (typical primary product in radical-type cyclohexane oxidation) supports a free-radical mechanism in this study.

As previously proposed for several homogeneous or heterogeneous M^n+1/n^ (e.g., V, Re, Fe, Cu or Au) catalytic systems [43,48,52,54,56,57] we can propose the following mechanism: copper-catalyzed decomposition of the peroxide ROOH (R = H or *^t^*Bu) leads to the oxygen-centered radicals ROO^•^ and RO^•^, upon oxidation by Cu(II) or reduction by Cu(I), respectively (reactions 1 and 2; in the case of Cu(I) compound **1**, reactions 1 and 2 occur in the reverse order, *i.e.*, first 2 and then 1). Water is believed to catalyze H^+^-shift steps towards the formation of RO^•^ [58,59,60]. Cycloalkyl radical Cy^•^ is then formed upon H-abstraction from cycloalkane CyH by RO^•^ (reaction 3). Reaction of Cy^•^ with dioxygen leads to CyOO^•^ (reaction 4), and CyOOH can then be formed upon H-abstraction from ROOH by CyOO^•^ (reaction 5) or upon reduction of the latter to CyOO^−^ by Cu(I) followed by protonation. Metal-assisted decomposition of CyOOH to CyO^•^ and CyOO^•^ (reactions 6 and 7) would then lead to cyclohexanol (CyOH) and cyclohexanone (Cy_-H_=O) products (reactions 8 and 9) [60].

Cu(II) + ROOH → ROO^•^ + H^+^ + Cu(I) (1)

Cu(I) + ROOH → RO^•^ + Cu(II) + HO^−^ (2)

RO^•^ + CyH → ROH + Cy^•^ (3)

Cy^•^+ O_2_ → CyOO^•^ (4)

CyOO^•^ + ROOH → CyOOH + ROO^•^ (5)

CyOOH + Cu(I) → CyO^•^ + Cu(II) + HO^−^ (6)

CyOOH + Cu(II) → CyOO^•^ + H^+^ + Cu(I) (7)

CyO^•^ + CyH → CyOH + Cy^•^ (8)

2CyOO^•^ → CyOH + Cy_-H_=O + O_2_ (9)

The initial availability of easily oxidized copper(I) to copper(II) species, or *vice versa* (easily reduced copper(II) to copper (I) species), to decompose the peroxide is crucial for this peroxidative oxidation, since the formation of the oxygen-centered radicals ROO^•^ and RO^•^ radicals is the key step for the occurrence of the C–H abstraction from the alkane.

Catalyst recyclability was investigated for up to four consecutive cycles for all the catalysts **1**–**3** on the oxidative media used for the conversion of cyclohexane. On completion of each stage, the products were analyzed as usually and the catalyst was recovered by filtration from the reaction mixture, thoroughly washed with acetonitrile and dried overnight at 60 °C. The subsequent cycle was initiated upon addition of new standard portions of all other reagents. The filtrate was analyzed relative to the presence of copper by atomic absorption spectroscopy and the hypothesis of catalyst leaching was excluded. Moreover, the filtrate was tested in a new reaction (by addition of fresh reagents), and no oxidation products were detected.

**Figure 5:**
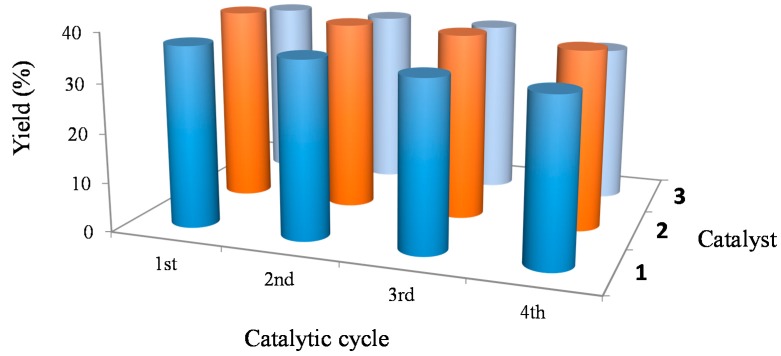
Effect of the catalyst recycling on the overall yield of the products from the cyclohexane oxidation catalyzed by **1**–**3**.

Figure 5 shows the recyclability of the systems: all were able to be reused while maintain almost the original level of activity after several consecutive reaction cycles (e.g., in a second, third and fourth run, the observed activity of **2** was 97%, 95%and 92% of the initial one) with a rather high selectivity to cyclohexanol and cyclohexanone.

In addition, to demonstrate the structure conservation, catalyst **2** was analyzed by FT-IR before and after the catalytic reaction, and no significant changes were detected. This suggests that **2** is a true heterogeneous catalyst, and no catalytically active species were released into the solution.

## 3. Experimental Section

### 3.1. General Information

All chemical reagents used in the experiments were purchased from commercial sources and no further purification was employed before using them for reactions. Microwave assisted synthesis of complex **1** was achieved in a focused microwave Discover reactor (150 W, CEM, Buckingham, UK), using a reaction tube of 10 mL capacity with a 13 mm internal diameter, fitted with a rotational system and an IR temperature detector (CEM). Infrared spectra (4000–500 cm^−1^) were recorded with a Tensor 27 (with MIR source, Zn–Se beam splitter and DLaTGS detector, Bruker, Bremen, Germany) of samples in KBr pellets. Elemental analyses were carried out with a Thermo-Flash 2000 elemental analyzer (Thermo Scientific, Waltham, MA, USA). The spectrophotometric measurements were performed on a Cary 100 UV-Vis spectrophotometer (Varian, Santa Clara, CA, USA) using a quartz cuvette with a path length of 1 cm. Powder X-ray diffraction patterns for complex **1** were recorded on a Smart Lab X-ray diffractometer (Rigaku, Wilmingtons, MA, USA). The X-rays used were of wavelength of 0.154 nm (CuK-α) produced using a sealed tube and detected using a linear counting detector (Scintillator NaI photomultiplier detector). 

X-ray crystallography: Single crystal X-ray structural studies of compound **1** were performed on a CCD (Oxford Diffraction, Agilent Technologies, Santa Clara, CA, USA) SUPER NOVA diffractometer. Data were collected at 150(2) K using graphite-monochromoated Mo Kα radiation (λ_α_ = 0.71073 Å). The strategy for the data collection was evaluated by using the CrysAlisProCCD software (Oxford Diffraction, Agilent Technologies). The data were collected by the standard phi-omega scan techniques and were scaled and reduced using CrysAlisPro RED software (Oxford Diffraction, Agilent Technologies). The structure was solved by direct methods using SHELXS-97 and refined by full matrix least-squares with SHELXL-97, refining on *F*^2^ [61,62]. The positions of all the atoms were obtained by direct methods. All non-hydrogen atoms were refined anisotropically. The remaining hydrogen atoms were placed in geometrically constrained positions and refined with isotropic temperature factors, generally 1.2*U_eq_* of their parent atoms. The crystal and refinement data are summarized in Table 3.

Gas chromatographic (GC) measurements were carried out using a FISONS Instruments GC 8000 series gas chromatograph with a FID detector and a capillary column (DB-WAX, column length: 30 m; internal diameter: 0.32 mm, FISONS, Markham, ON, Canada) and the Jasco-Borwin software (version 1.50, FISONS). The temperature of injection was 240 °C. The initial temperature was maintained at 100 °C for 1 min, then raised 10 °C/min to 180 °C and held at this temperature for 1 min. Helium was used as the carrier gas. GC-MS analyses were performed using a Perkin Elmer Clarus 600 C instrument (He as the carrier gas, Linde, Lisboa, Portugal). The ionization voltage was 70 eV. Gas chromatography was conducted in the temperature-programming mode, using a SGE BPX5 column (30 m × 0.25 mm × 0.25 µm, FISONS). Reaction products were identified by comparison of their retention times with known reference compounds, and by comparing their mass spectra to fragmentation patterns obtained from the NIST spectral library stored in the computer software of the mass spectrometer. 

**Table 3:** Crystallographic data and refinement details for **1**.

	1
Empirical formula	C_6_H_4_CuN_5_
Mr (g·mol^−1^)	209.68
Crystal system	Monoclinic
Space group	*P*2_1_/c
*a* (Å)	5.8169(2)
*b* (Å)	16.8804(6)
*c* (Å)	9.0264(5)
α (°)	90
β (°)	94.070
γ (°)	90
*V* (Å^3^)	884.08(7)
*Z*	4
*D*_calcd_ (mgm^−3^)	1.575
*F*(000)	416
GOF	1.259
Reflections collected/unique	5386/1546
Final R indices	R_1_ = 0.0355, wR_2_ = 0.1032
R indices (all data)	R_1_ = 0.0367, wR_2_ = 0.1036

### 3.2. Synthesis and Characterization of Complex [Cu(µ_4_-4-ptz)]_n_ (**1**)

A greenish brown mixture of CuCl_2_∙2H_2_O (51 mg, 0.3 mmol), NaN_3_ (39 mg, 0.6 mmol) and 4-cyanopyridine (125 mg, 1.2 mmol) in H_2_O and DMF (1 mL:6 mL) mixture was placed in a reaction tube that was irradiated with microwave radiation for 10 min at 130 °C. Cooling the reaction mixture to room temperature resulted in deposition on the walls of reaction tube of yellow colored, single crystals X-ray analysis quality crystals, and a bulk micro crystalline sample that precipitated out, whose powder X-ray diffraction (PXRD) patterns exactly matched simulated patterns from single crystal X-ray data (Figure 2) confirming the purity of the bulk sample. Yield = 53%, anal calc. for C_6_H_4_CuN_5_: C, 34.37, H, 1.92, N, 33.40, found: C, 34.5, H, 1.98, N, 33.43. IR (KBr): 1650(s), 1625(s) 1558(w), 1434(m), 1390(m), 1210(m), 1109(m).

### 3.3. Typical Procedures for the Catalytic Oxidation of Cycloalkanes and Product Analysis

The peroxidative oxidation reactions were typically carried out as follows: 0.1–20 μmol of the catalyst was added to 5.00 mmol of the cycloalkane, whereafter 10.00 mmol of 30% H_2_O_2_ (1.02 mL) or of 70% TBHP (688 μL) were added and the reaction solution was stirred for 10 h at r.t. and normal pressure. In the experiments with radical traps, CBrCl_3_ (5.00 mmol) or NHPh_2_ (5.00 mmol) was added to the reaction mixture. 

Catalyst recyclability was investigated, for up to four consecutive cycles. Each cycle was initiated after the preceding one upon addition of new typical portions of all other reagents. After completion of each run, the products were analyzed and the catalyst was recovered by filtration, washed with several portions of acetonitrile and dried in oven overnight at 60 °C.

The products analysis was performed as follows: 90 μL of cycloheptanone (internal standard), 10.00 mL of diethyl ether (to extract the substrate and the organic products from the reaction mixture) were added. The obtained mixture was stirred during 10 min and then a sample (1 μL) was taken from the organic phase and analyzed by gas chromatography (GC) by the internal standard method. Subsequently, an excess of solid triphenylphosphine was added to the final organic phase (to reduce the cyclohexyl hydroperoxide, if formed, to the corresponding alcohol, and hydrogen peroxide to water) and the mixture was analyzed again to estimate the amount of cyclohexyl hydroperoxide, following a method developed by Shul’pin [31,37,38,39]). For determination of oxygenate concentrations only data obtained after treatment of the reaction sample with PPh_3_ were used. Authentic samples of all oxygenated products were used to attribute the peaks in chromatograms. Blank tests indicate that no oxidation takes place in the absence of the Cu complex or the oxidant.

## 4. Conclusions

A novel microwave assisted methodology has been adopted to generate, within a few minutes, the Cu(I) based metal organic framework [Cu(µ_4_-4-ptz)]*_n_* (**1**). Copper based MOFs **1**–**3** act as catalysts for the mild and selective oxidation of cyclic alkanes in added solvent- and additive-free systems. A comparative study of their catalytic efficiency towards different cycloalkane substrates and oxidants has been performed. Furthermore, these heterogeneous greener catalytic systems allowed their easy recovery and reuse, at least for four consecutive cycles, maintaining over 90% of the initial activity and concomitant rather high selectivity. Moreover, the use of an aqueous medium at room temperature, without the requirement of an organic solvent, is a significant step towards the development of green catalytic systems in the field.

## Supplementary Materials

CCDC940213 contains the supplementary crystallographic data for compound **1**. These data can be obtained free of charge via http://www.ccdc.cam.ac.uk/conts/retrieving.html, or from the Cambridge Crystallographic Data Centre, 12 Union Road, Cambridge CB2 1EZ, UK; fax: (+44) 1223-336-033; or e-mail: deposit@ccdc.cam.ac.uk.
